# Preharvest sodium selenite treatments affect the growth and enhance nutritional quality of purple leaf mustard with abundant anthocyanin

**DOI:** 10.3389/fnut.2024.1447084

**Published:** 2024-10-23

**Authors:** Bin Wang, Xiao Yuan, Guang Wang, Yun-na Zhu, Run-chang Zhou, Hui-min Feng, Hai-bo Li

**Affiliations:** ^1^Guangdong Provincial Key Laboratory of Utilization and Conservation of Food and Medicinal Resources in Northern Region, College of Biology and Agriculture, Shaoguan University, Shaoguan, China; ^2^Guangdong Provincial Engineering and Technology Research Center of Special Fruit and Vegetables in Northern Region, Engineering and Technology Research Center of Shaoguan Horticulture in Shaoguan University, Shaoguan University, Shaoguan, China; ^3^College of Horticulture, South China Agricultural University, Guangzhou, China; ^4^Shaoguan Engineering and Technology Research Center of Leaf Mustard, Shaoguan University, Shaoguan, China; ^5^Mordern Seed Industry Research Institute of Renhua Danxia, Shaoguan, China

**Keywords:** preharvest treatment, selenium enrichment, leaf mustard, anthocyanin fortification, growth promotion

## Abstract

Both selenium (Se) and anthocyanins are crucial for maintaining human health. Preharvest Se treatments could promote anthocyanin biosynthesis and augment Se levels in vegetables, helping to combat Se deficiencies in dietary intake. However, it remains unknown whether preharvest Se treatment could balance growth and anthocyanin biosynthesis in plants and what the appropriate treatment concentration is. In this study, preharvest treatments with sodium selenite at varying concentrations (0, 5, 10, and 30 mg/kg) affect the growth and nutritional quality of purple leaf mustard (*Brassica juncea*) with abundant anthocyanins. Lower Se concentrations (≤10 mg/kg) of preharvest treatments enhanced photosynthesis, facilitated root system development, consequently elevated the biomass. Conversely, higher Se levels (≥30 mg/kg) reduced photosynthesis and biomass. The dosage-dependent effects of Se treatments were corroborated through seedlings cultivated in hydroponic conditions. Moreover, nearly all Se treatments elevated the contents of various nutrients in leaf mustard, particularly anthocyanin and organic se. These results suggest an overall enhancement in nutritional quality of leaf mustard plants. Furthermore, the application of 10 mg/kg Se significantly enhanced the activity of phenylalanine ammonia-lyase and upregulated the expression of 12 genes pivotal for anthocyanin biosynthesis, further demonstrating the fortified effects of Se enrichment on anthocyanins in leaf mustard. Low-level Se treatments resulted in heightened antioxidant activity (APX, CAT, and POD), mitigating reactive oxygen species induced by increasing Se content in tissues. The enhanced antioxidant activities may be beneficial for the normal growth of leaf mustard under Se stress conditions. In conclusion, our study demonstrated preharvest Se treatment at 10 mg/kg could balance the growth and anthocyanin biosynthesis in purple leaf mustard. This study offers valuable insights into anthocyanin fortification through Se enrichment methods in agricultural practices, ensuring that such fortification does not compromise leafy vegetable yield.

## Introduction

Selenium (Se) is a metalloid trace element that naturally occurs in nature, and is essential for maintaining human health (1). Deficiency of Se can negatively impact the human immune system, as well as increase the risk of cancers, inflammatory diseases, and neurodegenerative disorders (2, 3). Fortunately, Se deficiency in the human body can be addressed through the consumption of Se-enriched foods. Generally, organic Se species, such as Se-containing amino acids including selenocystine, Se-methylselenocysteine, and selenomethionine, have greater bioactivities and lower toxicity compared to inorganic Se for the human body (4). However, the process of enriching foods with organic Se is relatively difficult and expensive in practice. Plants have the ability to absorb both organic and inorganic Se from the soil and can convert inorganic Se to organic forms within their bodies (5). Therefore, Se-enriched agricultural products serve as an important source for individuals to fulfill their Se requirements and preserve optimal health.

However, Se deficiency in soil is a global issue (6), which poses significant challenges to agricultural productivity and human health. For instance, it has been reported that approximately 72% of the land area in China lacks Se (7). As a result, it is necessary to externally apply Se in order to enhance its availability in the soil and ultimately increase the Se levels in agricultural products. It has been reported that the application of Se in the soil has both positive and negative effects on plants. Low doses of Se treatments can enhance plant growth and resistance against various biotic and abiotic stresses (8). On the other hand, excessive accumulation of Se in plants can impede growth, lead to the generation of reactive oxygen species (ROS), damage the photosynthesis system, disrupt nutrient absorption and transport, and ultimately cause genotoxicity in plants (9). For instance, low-level (5 μM) Se treatment could promote the growth of arsenic (As)-hyperaccumulator *Pteris vittata* under normal and As stress conditions (10). However, high Se concentrations (≥24 μM) reduced photosynthetic efficiency and antioxidant enzyme activity, disturbed carbon and nitrogen metabolism in apple plants (11). Although the effects of Se on plant growth have been extensively documented, more investigations are needed, particularly in those characteristic vegetable species with high content of bioactive compounds such as anthocyanin, to guide production practices due to plant species diversity.

Se application not only affects the growth and development of plants, but also enhances the nutritional quality by inducing the biosynthesis of secondary metabolites (12). The contents of secondary metabolites including phenolic compounds, carotenoids, flavonoids, proteins, glycosides, glutathione and vitamins were significantly increased after Se supplementation (13–15). Among them, anthocyanins are water-soluble pigments, garnering significant attention for their numerous health benefits in agricultural products (16), especially in fresh fruit and vegetables. Previous studies have demonstrated that anthocyanins possess potent antioxidant, anti-inflammatory, anticancer, antidiabetic, antihyperlipidemic, and antiulcer properties (17–19). Moreover, they have been associated with improved cardiovascular health, enhanced immune function, and the prevention of age-related diseases (20, 21). Therefore, it can be reasonably inferred that enhancing anthocyanins could amplify the beneficial biological activities of pigmented fruit and vegetables.

Recent studies have shown that Se supplement in soils can stimulate anthocyanin biosynthesis and accumulation during grain development in several crop species (22). It has been reported that Se treatment could promote anthocyanin accumulation in radish sprouts (*Raphanus sativus*) by enhancing photosynthesis and sucrose transport (23). In wheat seeds, Se biofortification increased the contents of Se and anthocyanin, and genes responsible for anthocyanin biosynthesis were significantly upregulated after Se treatment (24). Pigmented wheat cultivars exhibited higher Se efficiency absorption and the same transcription factors, such as 2R-MYB and bHLH, control both the Se flow direction and anthocyanin biosynthesis (25). Notably, anthocyanins are also responsible for the vibrant colors observed in plants, which typically range from red to purple and blue (26). The anthocyanins in fruit and vegetables not only enhances consumers’ appeal but also brings significant economic benefits (23). While fruit, seeds, and flowers of plants are reproductive organs and the development does not depend the photosynthesis for energy production, anthocyanin fortification by Se treatment in these organs does not result in a significant decrease in biomass. However, excessive accumulation of anthocyanins in vegetative organs, such as leaves, can intensify leaf coloration, potentially reducing photosynthetic efficiency, and thus leading to a delayed growth (27). Hence, it is essential to investigate whether and how Se-induced anthocyanin biosynthesis in leaves impacts the growth and yield of anthocyanin-enriched vegetables.

*Brassica* vegetables are one of the most widely consumed horticultural crops worldwide, known for their diverse array of nutrients (28). In the past few years, our research team has successfully developed a novel purple leaf mustard (*Brassica juncea*) variety named “Zifei,” which has abundant anthocyanins in the leaf. Furthermore, it has been demonstrated that anthocyanins in *Brassica* vegetable leaf are mainly accumulated at the adaxial epidermis of leaves (29), and the accumulation of anthocyanins in leaf mustard leaves results in reduced photosynthetic efficiency and biomass (30). Therefore, it is of great interest to investigate whether preharvest Se treatment could balance growth and anthocyanin biosynthesis and what the appropriate concentration is in leafy vegetables. Therefore, the main objectives of this study are: (a) to examine the changes in phenotype of purple leaf mustard when exposed to different concentrations of Se treatments before harvest; (b) to assess the influence of Se treatment on the nutritional quality of purple leaf mustard, especially regarding anthocyanin biosynthesis. The results of this study enriched the anticipated relationships between Se biofortification and anthocyanin biosynthesis in plants.

## Materials and methods

### Leaf mustard cultivation and sodium selenite treatment

The seeds of leaf mustard (‘Zifei’, a novel hybrid cultivar with high anthocyanin content in leaves) were initially sown in a seedling tray containing a mixture of peat and perlite in a 1:1 ratio. After 14 days of germination, seedlings having consistent growth were transplanted into square plates measuring 8 × 8 cm (height × width). For the investigation of preharvest sodium selenite treatment effects, about 40-d-old leaf mustard plants were employed. The seedlings were irrigated with the 1‰ (m/v) Hyponex solution (Chembase, Japan) and cultivated in an artificial climate room maintained at a temperature of 25°C with a 12-h light (1.5 × 104 ± 250 lx)/dark cycle (31). Different concentrations of sodium selenite (0, 5, 10, and 30 mg/kg on a dry weight basis of substrates) were administered to the substrates 7 days before harvest. Each treatment group consisted of 48 plants, with three replications, each comprising 16 plants. Subsequently, the leaf mustards were harvested to assess the impacts of preharvest Se treatments on the growth and nutritional quality.

### Analysis of biomass and root system of leaf mustard plants

The fresh and dry weight of different parts (leaf and root) of each individual plant were measured separately using an electronic scale. Prior to determining the dry weight, the seedlings were dried until a constant weight was achieved. The shoot diameter (at the junction between the leaf and root) of the leaf mustard was assessed using a Vernier caliper. The root system of the leaf mustard was scanned and analyzed using a MICROTEK root analysis scanner (MRS-9600 TFU2L, China) (32), and the data included root length, projected area of root system, and root tip number.

### Measurement of photosynthetic parameters

At 7 days of Se treatment, the 3rd – 4th main stem leaves from the bottom of the plant were selected for measurements of photosynthetic parameters (Pn, net photosynthetic rate; Tr, transpiration rate; C leaf, stomatal conductance; CO_2_ int., intercellular CO_2_ concentration) and chlorophyll fluorescence parameters (*F*o, *Fv*, *Fm*, *Fv/Fm*) using a portable photosynthesis system (Yaxin-1105, China). Before the measurements of chlorophyll fluorescence parameters, leaf mustard seedlings were held at dark environment for 20 min for dark adaptation. Measurements were taken between 9:00 and 11:30 a.m., and repeated three times for each leaf.

Relative chlorophyll contents (SPAD) in leaves was determined using a portable plant nutrient analyzer (TYS-4N, China) (33). To extract chlorophyll and carotenoid pigments, 1.0 g of fresh samples was soaked in 95% ethanol for 48 h. The mixture was then centrifuged at 11,000 × *g* at 4°C for 10 min. The supernatants were then analyzed at 665, 649 and 470 nm using a UV spectrophotometer (T2602, YOKE Instrument, China) to measure the amounts of chlorophylls and carotenoids (34).

### Determination of leaf color and total anthocyanin content (TAC) in mustard plant leaves

The color parameters (L*, a*, b* and *ΔE*) on the obverse side of leaf were measured with a CR-400 Chroma meter (KONICA MINOLTA, Japan). Total anthocyanin in leaves was extracted with methanol-HCl method. In total, 1.0 g of fresh samples were soaked in 5 mL of 1% (v/v) methanol-HCl for 24 h in dark environment. The absorbance of extract was concurrently measured at 530, 620, and 650 nm. Total anthocyanin content (TAC) was quantified according to the following formula: TAC = (OD530 − OD620) − 0.1 × (OD650 − OD620) (35).

### Assay of total Se contents in mustard plant leaves and roots

Total Se contents in leaves and roots of leaf mustard seedlings were determined according to the China National Standard (GB 5009.93–2017). In total, 0.5 g of dry samples were ground into powder and digested with 10 mL of mixture of nitric acid and perchloric acid (10:1). The digested solution was reconstituted to 10 mL with 100 g/L potassium ferricyanide solution. Total Se contents were determined using an atomic fluorescence spectrometer (HGF-V9, Haiguang Instrument, China) (36). The result was expressed as milligram per kilogram dry weight (mg/kg DW).

### Determination of total soluble solids (TSS), glutathione (GSH), and total soluble protein (TSP) contents

For TSS determinations, 100 g of fresh samples from three independent plants were homogenized and filtered. The 0.2 mL of filtrates were used to measure TSS contents using a handheld digital refractometer (Atago, Japan). GSH contents in leaf mustard plants were determined using a test kit (Sangon Biotech, China) following the manufacturer’s instructions (37).

The TSP contents were determined with the Coomassie brilliant blue (CBB) method (38). Briefly, 1.0 g of leaf samples was homogenized into 5 mL of 0.5 mM phosphate buffer (pH 7.0). The homogenates were centrifuged at 11,000 × *g* at 4°C for 10 min. For the measurement of TSP content, 0.2 mL of the supernatants were mixed with 5 mL of 0.1 g/L CBB and reacted at room temperature for 15 min. The absorbance was recorded at 595 nm.

### Determination of ascorbic acid, free amino acid and total phenolic contents

For this, 1.0 g of fresh samples was homogenized with 5 mL of 10 mmol/L trichloroacetic acid. The homogenates were centrifuged at 11,000 × *g* for 10 min and then were used to measure ascorbic acid content (AAC) according to the methods described by Tan et al. (39). Briefly, 1.0 mL of supernatants, 1 mL of 10 mmol/L trichloroacetic acid, 1 mL of ethanol, 0.5 mL of 0.4% (v/v) phosphoric acid, 1 mL of 5 g/L 4,7-Diphenyl-4,10-Phenanthroline and 0.5 mL of 0.2 mmol/L FeCl_3_ were mixed in a tube. The absorbance of mixtures was monitored at 543 nm.

Free amino acid (FAA) content was determined using the hydrated ninhydrin method as described by Liu et al. (11). In total, 1.0 g of leaf samples were homogenized with 5 mL of ultrapure water and subjected to a boiling water bath for 20 min. The resulting homogenates were filtered to obtain the crushed extracts for FAA analysis. The reaction system consisted of 3 mL, which included 0.5 mL of the crushed extracts, 2 mL of 5 mmol/L cyanate buffer, and 0.5 mL of 10 mmol/L ninhydrin. The mixed solutions were allowed to react at room temperature for 1 h, and the absorbance was measured at 570 nm.

Total phenolic content (TPC) was extracted and quantified using a modified version of the method previously used by Yuan et al. (40). In summary, 1.0 g of leaf samples were homogenized with 5 mL of 1% (v/v) HCl-methanol, extracting at 25°C for 2 h. The extracts were then centrifuged at 4°C at 11,000 × *g* for 10 min. For the analysis, 0.5 mL of the supernatants, 1.5 mL of 1 mol/L sodium carbonate, and 1.0 mL of Folin–Ciocalteu reagent were sequentially added into a new tube, mixed thoroughly and incubated at 25°C for 1 h in dark. The absorbance of the resulting solution was measured at 765 nm. TPC was calculated with a standard curve generated from varying concentrations of gallic acid.

### qRT-PCR analysis

Total RNA of mustard plants was extracted using a RNAprep extraction Kit (DP441, Tiangen, China) according to the kit instructions. RNA integrity and concentration were checked using a Nano Photometer (Nano 300, ALL SHENG, China). The cDNA was synthesized using a cDNA Synthesis Kit (11123ES60, Yeasen, China) following the instructions. Quantitative real-time PCR (qRT-PCR) was performed using a real-time PCR system (CFX Opus 384, Bio-Rad, United States) and a qPCR analysis kit (11201ES03, Yeasen, China) (41). The primer sequences used for qRT-PCR analysis were listed in Supplementary Table S1.

### Measurement of hydrogen peroxide and malondialdehyde content in leaf mustard plants

Hydrogen peroxide (H_2_O_2_) and alondialdehyde (MDA) in leaf mustard samples were extracted with normal saline. For the extraction of crude extracts, 1.0 g of leaf samples was homogenized with 5 mL of normal saline. The extracts were then centrifuged at 4°C at 11,000 × *g* for 10 min and used for H_2_O_2_ and MDA content measurements. The levels of H_2_O_2_ and MDA were quantified using commercial kits, following the instructions provided by the manufacturer (42).

### Enzyme activity determination in leaf mustard plants

As for the preparation of crude enzymes, 1.0 g of samples were homogenized in 5 mL of 0.5 mM phosphate buffer (pH 7.0) containing 1% PVP. The homogenates were then centrifuged at 11,000 × *g*, 4°C for 15 min, and the supernatants were collected for enzyme activity measurements.

For phenylalanine ammonia-lyase (PAL) activity measurement, 0.5 mL of each solution including supernatants, 5 mmol/L dithiothreitol and 20 mmol/L L-phenylalanine were first mixed and added up to a total volume of 3 mL with 0.1 mmol/L sodium borate (pH 8.8). The mixed solutions were incubated at room temperature (25°C) for 2 h, and the absorbance of the reaction was determined at 290 nm.

The activities of peroxidase (POD) and catalase (CAT) were measured according to the methods introduced by Wang et al. (42). The POD activity was assayed by measuring the oxidation rate of guaiacol at 470 nm, while the CAT activity was determined by monitoring the decomposition rate of hydrogen peroxide at 240 nm. Additionally, the ascorbate peroxidase (APX) activity was measured using both ascorbic acid and hydrogen peroxide as substrates at 290 nm (37).

### Phenotype analysis of leaf mustard seedlings under Se stress condition

To confirm the effects of sodium selenite treatments on the growth of leaf mustard, a separate experiment was conducted using seedlings as the experimental material (Supplementary Figure S1). Seedlings were planted in a 1‰ (m/v) Hyponex solution containing sodium selenite, with final concentrations of 0, 5, 10, and 30 mg/L. The seeds were sown and incubated in 15 cm diameter culture dishes for 7 days at 25°C under 12-h light/dark cycles. The germination rate (%) was evaluated 2 days after sowing, while fresh weight, hypocotyl length, and relative electrolyte leakage (REL) of the seedlings were measured after 7 days of exposure to Se stress. Each treatment group consisted of three replicates of 60 seedlings for phenotypic analysis.

To determine the REL, three seedlings from each treatment were immersed into 25 mL of distilled water at room temperature for 2 h. The electrical conductivity (EC) was measured using a handheld conductance bridge (DDB-303A, INESA, China). Total EC was measured again after boiling the tube for 30 min, and REL was expressed as a percentage of the total EC (37).

### Reactive oxygen species (ROS) staining in leaf mustard seedlings under Se stress conditions

To investigate the effects of varying concentrations of Se treatments on ROS contents in seedlings, leaf mustard seeds were incubated in different concentrations of Se solutions (0, 5, 10, and 30 mg/L) for 7 days. The whole seedlings were used to stain the levels of two major ROS, H_2_O_2_ and superoxide anion (O_2_^-.^). H_2_O_2_ and O_2_^-.^ in seedlings were stained with the nitro-blue tetrazolium (NBT) and 3,3’-Diaminobenzidine (DAB) reagents (43).

### Statistical analysis

The experiment was conducted using a random design, and all values were reported as mean ± standard deviation (SD) with a sample size of 3 (*n* = 3). Statistically significant differences among treatments were compared by the student’s *t*-test and/or one-way ANOVA in the SPSS software (version 22.0), with a significant level set at *p* < 0.05. To avoid Type I errors, the Bonferroni correction was considered for conducting the significance analysis. Statistical differences were indicated using different letters.

## Results

### Effects of varying concentrations of Se treatments on the biomass of leaf mustard plants

As shown in Table 1, the growth of purple leaf mustard was affected by varying concentrations of sodium selenite treatments. The shoot diameters, as well as the fresh and dry weight of roots, leaves and the whole plant of leaf mustard exhibited a similar trend following the various Se treatments. As the Se concentration increased gradually, the values also increased before declining (Table 1). Treatment with 5 mg/kg Se slightly enhanced shoot diameters and the fresh and dry weights of the plant, though there was no significant difference compared to the control (0 mg/kg). On the other hand, the 10 mg/kg Se treatment led to significant increases in biomass, while the 30 mg/kg Se treatment resulted in a significant reduction in leaf mustard biomass compared to the control. These results suggest that an appropriate concentration of Se treatment can enhance the growth of leaf mustard, indicating that preharvest Se application has the potential to improve purple leaf mustard yield.

**Table 1:** The biomass of leaf mustard affected by different concentrations of sodium selenite preharvest treatments.

Se concentration	Fresh weight of leaf (g)	Fresh weight of root (g)	Fresh weight of whole plant (g)	Dry weight of leaf (g)	Dry weight of root (g)	Dry weight of whole plant (g)	Shoot diameter (cm)	Root total length (×10^3^ cm)	Projected area of root (×10^3^ cm^2^)	Root tip number (*D* ≤ 0.5 mm)
0 mg/kg	40.05 ± 4.31b	8.91 ± 2.07ab	47.60 ± 0.95b	0.52 ± 0.03b	1.29 ± 0.47b	1.83 ± 0.42b	0.73 ± 0.06b	14.61 ± 1.73b	11.96 ± 0.43b	457.00 ± 50.20b
5 mg/kg	40.45 ± 1.33b	10.00 ± 0.04b	55.33 ± 8.18ab	0.49 ± 0.01c	1.71 ± 0.24ab	2.54 ± 0.12ab	0.93 ± 0.23ab	16.63 ± 1.22b	12.47 ± 0.49b	500.00 ± 14.00b
10 mg/kg	50.66 ± 5.73a	10.54 ± 0.16a	59.72 ± 7.25a	0.73 ± 0.06a	2.10 ± 0.16a	2.59 ± 0.34a	1.14 ± 0.15a	20.04 ± 1.14a	14.09 ± 0.96a	583.00 ± 51.64a
30 mg/kg	21.37 ± 0.67c	4.28 ± 0.27c	25.17 ± 0.94c	0.34 ± 0.03d	0.72 ± 0.05c	1.25 ± 0.08c	0.53 ± 0.06c	10.40 ± 0.69c	9.24 ± 0.92c	399.67 ± 14.01c

Sodium selenite was added to the soil at 7 days before harvest. The data is presented as means ± standard error from three replicates. Statistical differences (*p* < 0.05) are denoted by different letters accompanying the data.

Root development plays a vital role in plant growth, prompting us to examine the effects of Se treatment on root system. As anticipated, the 10 mg/kg Se treatment notably increased root length, projected area of root system, and root tip number, whereas the 30 mg/kg Se treatment decreased these parameters compared to the control (Table 1).

### Effects of varying concentrations of Se treatments on the photosynthesis of mustard plant leaves

**Figure 1:**
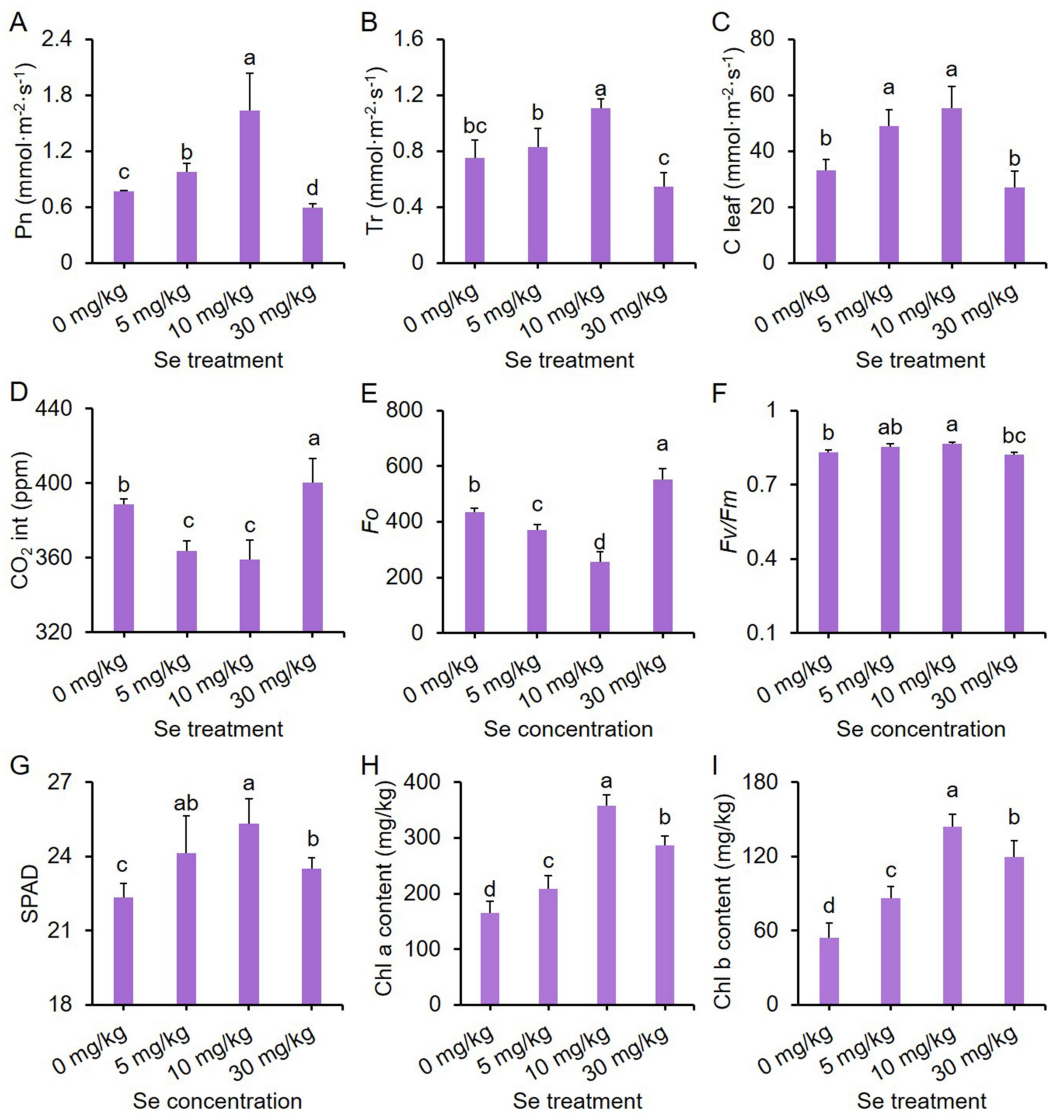
Effects of preharvest Se treatments with different concentrations on the photosynthesis of purple leaf mustard plant. **(A)** Net photosynthetic rate (Pn); **(B)** transpiration rate (Tr); **(C)** stomatal conductance (C leaf); **(D)** intercellular CO_2_ (CO_2_ int); **(E)** chlorophyll fluorescence parameter *Fo*; **(F)** chlorophyll fluorescence parameter *Fv/Fm*; **(G)** relative chlorophyll content (SPAD); **(H)** chlorophyll a content; **(I)** chlorophyll b content. Different concentrations of sodium selenite (0, 5, 10, and 30 mg/kg) were added to the soil at 7 days before harvest. The data is presented as means ± standard error from three replicates. Statistical differences (*p* < 0.05) are denoted by different letters above the bars.

Four major photosynthetic parameters, including net photosynthetic rate (Pn), transpiration rate (Tr), stomatal conductance (C leaf) and intercellular CO_2_ (CO_2_ int), were measured in the leaves to evaluate the effects of preharvest Se treatment on photosynthesis. The photosynthetic parameters of purple leaf mustard were influenced by varying concentrations of Se treatments. With increasing Se treatment concentration, the values of Pn, Tr, and C leaf exhibited a pattern of initially increasing and then decreasing, while the intercellular CO_2_ (CO_2_ int) showed the opposite trend (Figures 1A–D). The Se treatment at a concentration of 10 mg/kg resulted in the highest values for Pn, Tr, and C leaf, which were 111.21, 47.56, and 67.76% higher than the control, respectively. Additionally, the values of CO_2_ int. for the 10 mg/kg Se treatment were significantly lower than those of the control. In contrast, the Pn values for the 30 mg/kg Se treatment were significantly lower compared to the control, while the CO_2_ int. values for the 30 mg/kg Se treatment were significantly higher (Figures 1A–D). These results, in conjunction with other literatures, indicate that an appropriate Se treatment could enhance photosynthetic efficiency, while high concentrations of Se treatment may lead to photo inhibition in the leaves (11).

In order to delve deeper into the impact of Se on the photosynthesis of leaf mustard, we also analyzed chlorophyll fluorescence parameters and chlorophyll pigment contents. The parameter *Fo* represents the minimum fluorescence yield, indicating the baseline fluorescence level when all PSII reaction centers are open. On the other hand, the parameter *Fv/Fm* signifies the maximum quantum efficiency of PSII, with higher values indicating enhanced photosynthetic efficiency (44). *Fo* values showed a trend of decreasing and then increasing with rising Se concentration, with the 10 mg/kg Se treatment displaying the lowest *Fo* value (Figure 1E). On the other hand, *Fv/Fm* exhibited a completely opposite trend to *Fo* under varying Se treatments (Figure 1F). Nevertheless, all Se treatments significantly boosted chlorophyll pigment contents compared to the control, including relative chlorophyll content (SPAD), chlorophyll a and chlorophyll b contents, with the Se treatment at 10 mg/kg concentration yielding the highest chlorophyll contents (Figures 1G–I). These results suggest that the influence of Se treatment on purple leaf mustard growth is possibly associated with the efficiency of light utilization, rather than a reduction in the biosynthesis of chlorophyll pigments in purple leaf mustard.

### Se treatments deepen the color of purple leaf mustard plant

To investigate the impact of Se treatment on the color of purple leaf mustard plants, we examined the color of the leaf surface and the levels of chromogenic substances, including anthocyanin and carotenoids. Within color parameters, L^*^ signifies the lightness of a color, while a^*^ and b^*^ gauge its position on the green-red and blue-yellow axes, respectively. In addition, *ΔE* denotes the overall color difference (40).

**Figure 2:**
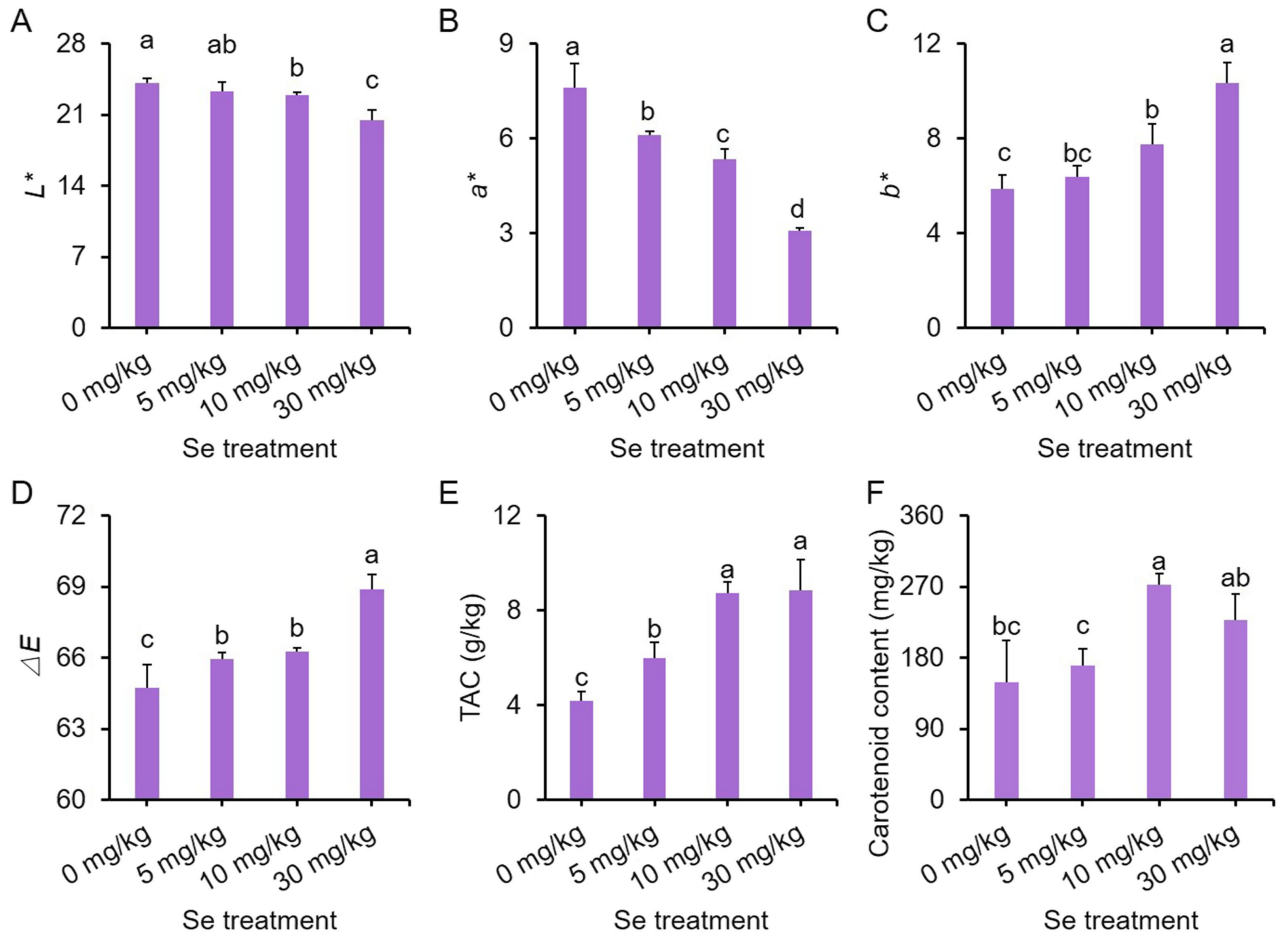
Effects of preharvest Se treatments with different concentrations on leaf surface color of purple leaf mustard plants. **(A)** L*; **(B)** a*; **(C)** b*; **(D)** chromatism *ΔE*; **(E)** total anthocyanin content (TAC); **(F)** carotenoid content. Different concentrations of sodium selenite (0, 5, 10, and 30 mg/kg) were added to the soil at 7 days before harvest. The data is presented as means ± standard error from three replicates. Statistical differences (*p* < 0.05) are denoted by different letters above the bars.

Both L* and a* values declined with increasing Se concentration, while b* and *ΔE* values had the opposite trend. Se treatments at concentrations higher than 5 mg/kg notably decreased L* and a* values but increased b* and *ΔE* values compared to the control (Figures 2A–D). In comparison to the control, all Se treatments exhibited significantly higher total anthocyanin content (TAC), with the content increasing as the Se concentration rose (Figure 2E). Furthermore, Se treatments at 10 and 30 mg/kg concentrations led to higher carotenoid content relative to the control (Figure 2F). These results collectively suggest that Se treatment may promote the accumulation of pigments, thereby intensifying the coloration.

We also assessed the ratio of total chlorophyll to TAC to investigate whether the buildup of pigments, particularly dark anthocyanin, impacts the photosynthesis of the leaf mustard plants. All Se treatments enhanced total chlorophyll contents, with the 10 mg/kg Se treatment displaying the highest content (Supplementary Figure S2A). However, only the 10 mg/kg Se treatment showed a higher ratio of total chlorophyll to TAC compared to the other Se treatments and the control (Supplementary Figure S2B). These results imply that the heightened pigment levels may indeed influence photosynthesis.

### Se treatments improve the nutritional quality of leaf mustard plant

**Figure 3:**
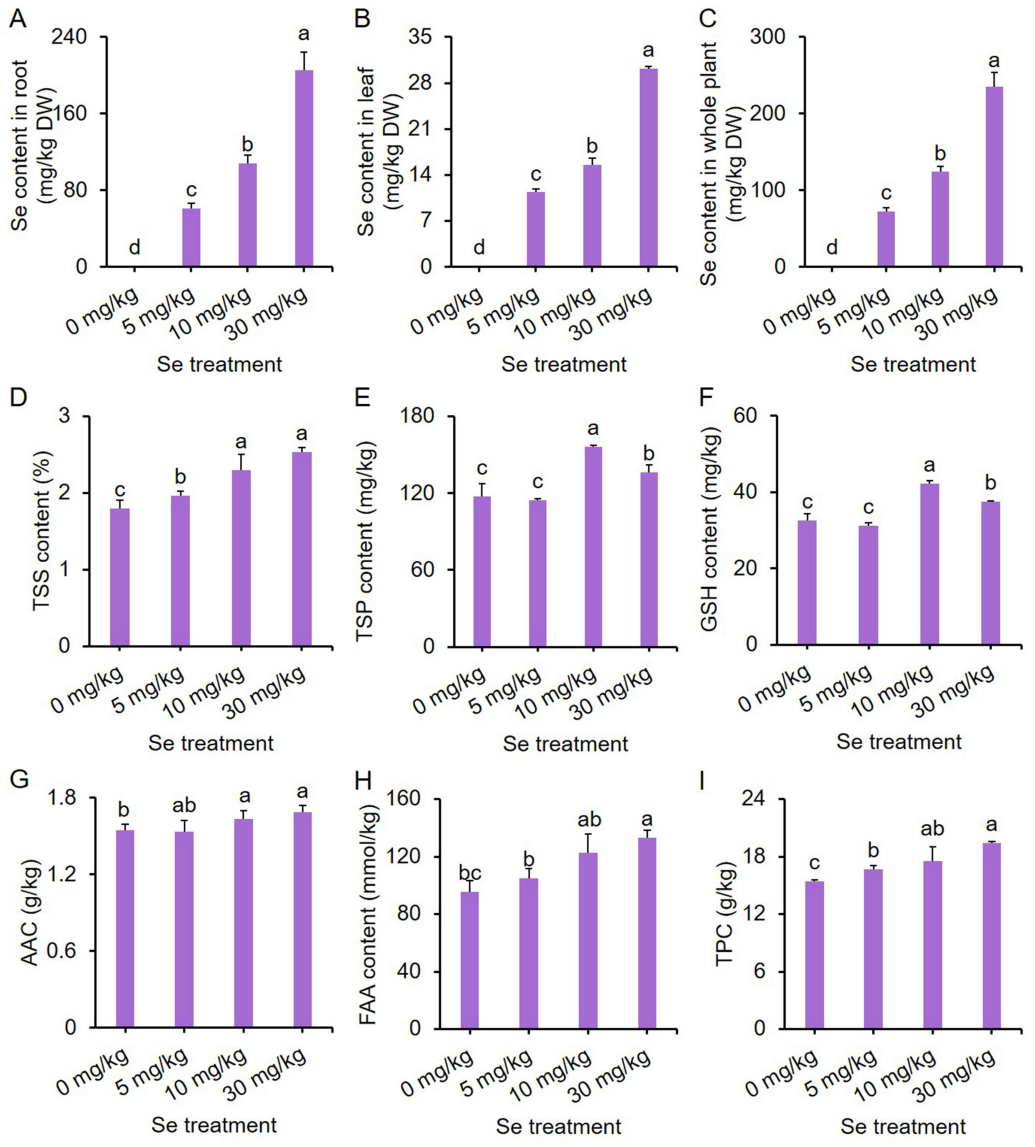
Effects of preharvest Se treatments with different concentrations on nutritional quality of purple leaf mustard plant. **(A)** Total selenium (Se) content in root; **(B)** total Se content in leaf; **(C)** total Se content in the whole plant; **(D)** total soluble solid (TSS) content; **(E)** total soluble protein (TSP) content; **(F)** glutathione (GSH) content; **(G)** ascorbic acid content (AAC); **(H)** free amino acid (FAA) content; **(I)** total phenolic content (TPC). Different concentrations of sodium selenite (0, 5, 10, and 30 mg/kg) were added to the soil at 7 days before harvest. The data is presented as means ± standard error from three replicates. Statistical differences (*p* < 0.05) are denoted by different letters above the bars.

To assess the ability of purple leaf mustard to enrich Se, total Se levels in both leaves and roots were determined. Total Se contents in both leaves and roots increased with the concentration of Se treatment, with roots accumulating more Se (Figures 3A–C), indicating a high enrichment efficiency of purple leaf mustard for Se.

Furthermore, the total soluble solid (TSS) content increased with increasing Se treatment concentrations, with all Se treatments showing higher TSS content compared to the control (Figure 3D). Different Se treatment intensities elicited similar trends in total soluble protein (TSP) and glutathione (GSH) contents (Figures 3E,F). Both the 10 and 30 mg/kg Se treatments resulted in significantly higher TSP and GSH contents, with the 10 mg/kg Se treatment exhibiting the highest content.

For ascorbic acid content (AAC) and free amino acid (FAA) content, only the 10 mg/kg and 30 mg/kg Se treatments significantly enhanced AAC and FAA content as compared to the control (Figures 3G,H). In contrast, all Se treatments resulted in a significantly higher total phenolic content (TPC) than the control (Figure 3I). These results clearly suggest that preharvest Se treatment could improve the nutritional quality of leaf mustard.

### Se treatment upregulates the expression of key genes in anthocyanin biosynthesis pathway

**Figure 4:**
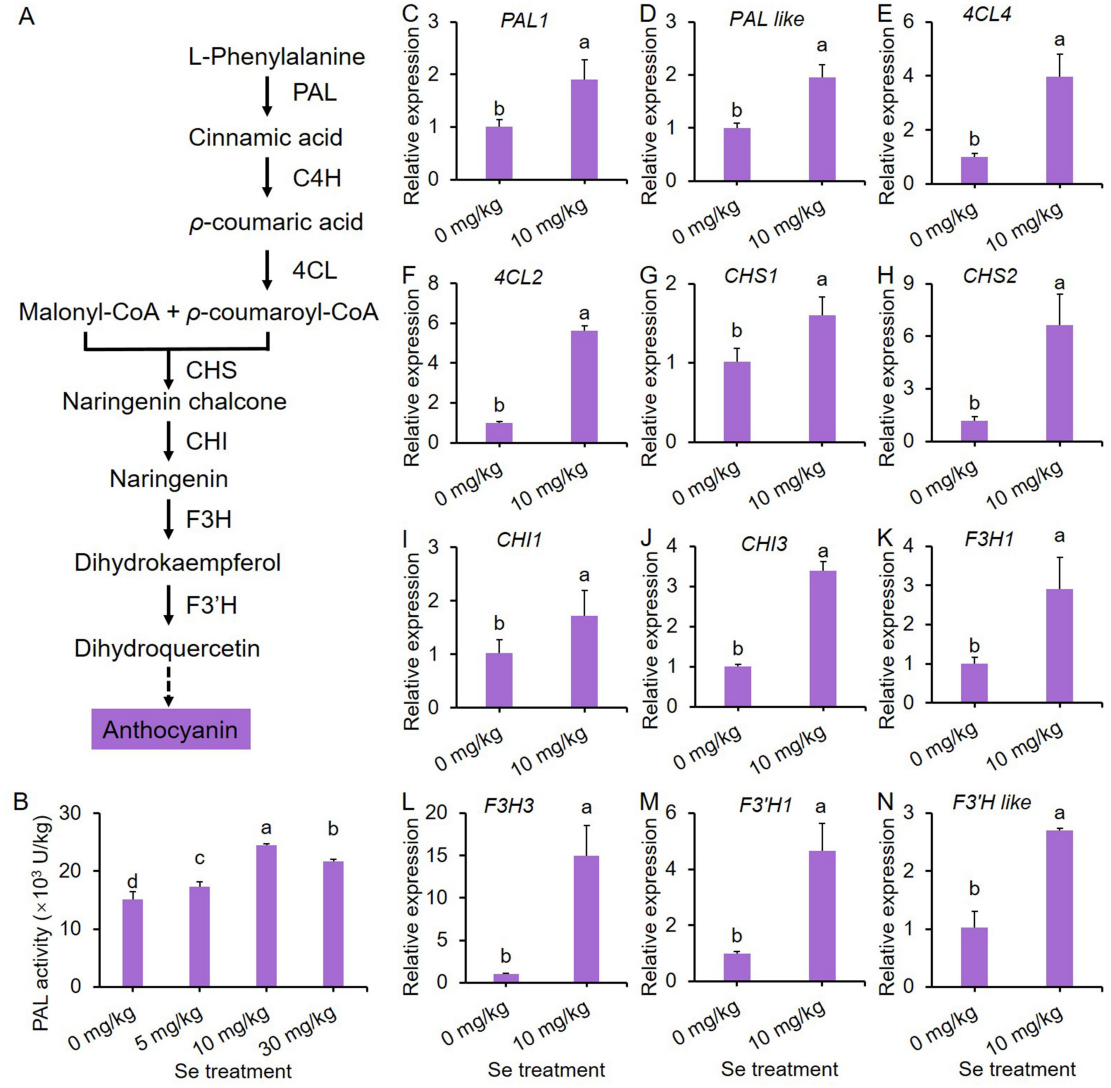
10 mg/kg Se treatment induces the biosynthesis of anthocyanin through the phenylpropanoid pathway in purple leaf mustard plant. **(A)** A simplified pathway for anthocyanin biosynthesis; **(B)** phenylalanine ammonia-lyase (PAL) activity; **(C–N)** the gene expression profiles of key enzymes responsible for anthocyanin biosynthesis in the phenylpropanoid pathway. Different concentrations of sodium selenite (0, 5, 10, and 30 mg/kg) were added to the soil at 7 days before harvest. The data is presented as means ± standard error from three replicates. Statistical differences (*p* < 0.05) are denoted by different letters above the bars.

Anthocyanin is synthesized through the phenylpropanoid pathway (45). As Se treatments intensified the purple color and enhanced total anthocyanin content (TAC) in purple leaf mustard, we investigated the impact of Se treatment on protein activity and gene expression of key enzymes responsible for anthocyanin biosynthesis. A simplified pathway for anthocyanin biosynthesis in plants is illustrated in Figure 4A.

In Figure 4B, it is evident that Se treatments increased PAL activity, which is the initial enzyme in the anthocyanin biosynthesis pathway, compared to the control. Furthermore, the Se treatment at 10 mg/kg exhibited the highest PAL activity. Consequently, this concentration of Se treatment was chosen as a representative to study the effects of Se treatments on gene expression in the phenylpropanoid pathway.

A total of 14 representative genes were selected to analyze their expression profiles. As depicted in Figures 4C–N, the expression of almost all genes was significantly up-regulated by Se treatment, with the exception of two *C4H* genes (Supplementary Figure S3). These results show that Se treatment stimulates anthocyanin biosynthesis by inducing the gene expression of key enzymes in the phenylpropanoid pathway.

### Effects of varying concentrations of Se treatments on ROS metabolism in leaf mustard plants

Antioxidant enzymes are crucial for supporting plant growth under abiotic stress and play essential roles in maintaining a balance between the production and breakdown of reactive oxygen species (ROS) (46). As such, we conducted an investigation to examine the impact of Se treatment on ROS metabolism in leaf mustard plants.

**Figure 5:**
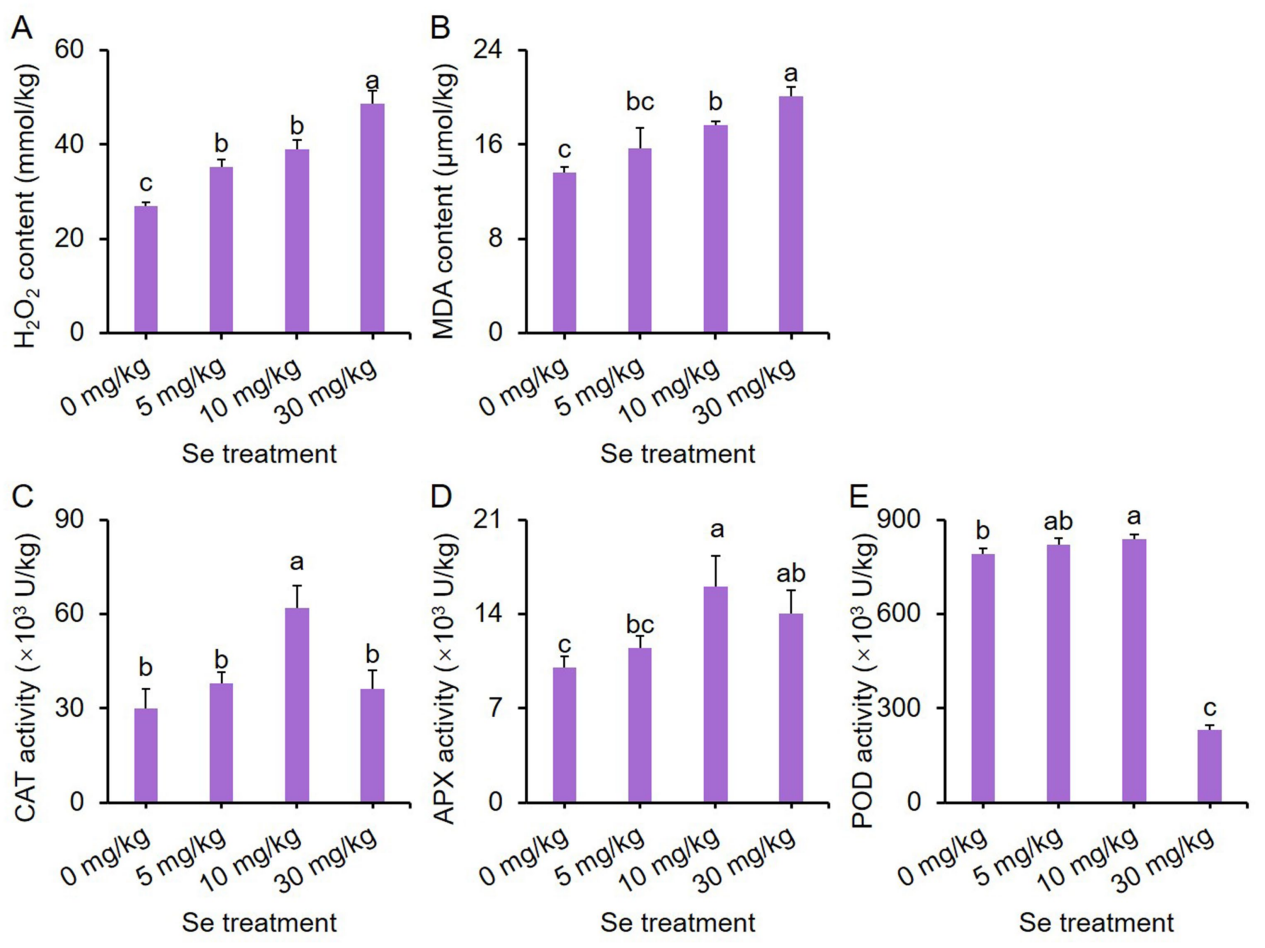
Effects of preharvest Se treatments with different concentrations on reactive oxygen species (ROS) metabolism in purple leaf mustard plants. **(A)** Hydrogen peroxide (H_2_O_2_) content; **(B)** malondialdehyde (MDA) content; **(C)** catalase (CAT) activity; **(D)** ascorbate peroxidase (APX) activity; **(E)** peroxidase (POD) activity. Different concentrations of sodium selenite (0, 5, 10, and 30 mg/kg) were added to the soil 7 days before harvest. The data is presented as means ± standard error from three replicates. Statistical differences (*p* < 0.05) are denoted by different letters above the bars.

The levels of hydrogen peroxide (H_2_O_2_) and malondialdehyde (MDA) increased with the concentration of Se treatment. All Se treatments showed significantly higher H_2_O_2_ contents compared to the control (Figure 5A), while only the 10 and 30 mg/kg Se treatments resulted in elevated MDA contents (Figure 5B). These results suggest that Se treatment induces oxidative stress in purple leaf mustard.

In terms of catalase (CAT) activity, only the 10 mg/kg Se treatment led to enhanced activity (Figure 5C). The ascorbate peroxidase (APX) activity for both the 10 and 30 mg/kg Se treatments was notably higher than that of the other treatments, with the 10 mg/kg Se treatment displaying the highest APX activity (Figure 5D). Additionally, the peroxidase (POD) activity was highest with the 10 mg/kg Se treatment and lowest with the 30 mg/kg Se treatment (Figure 5E). These results indicate that Se treatment can influence the activity of antioxidant enzymes.

### Effects of varying concentrations of Se treatments on the growth performance of leaf mustard seedlings

The application manner of Se may affect plant growth. The addition of optimal concentration of Se element in soil has been shown to improve the growth and nutritional quality of leaf mustard plants in this study. To assess whether Se treatment yields consistent effects on leaf mustard under different cultivation methods, an additional experiment was conducted using seedlings in a hydroponic system.

**Figure 6:**
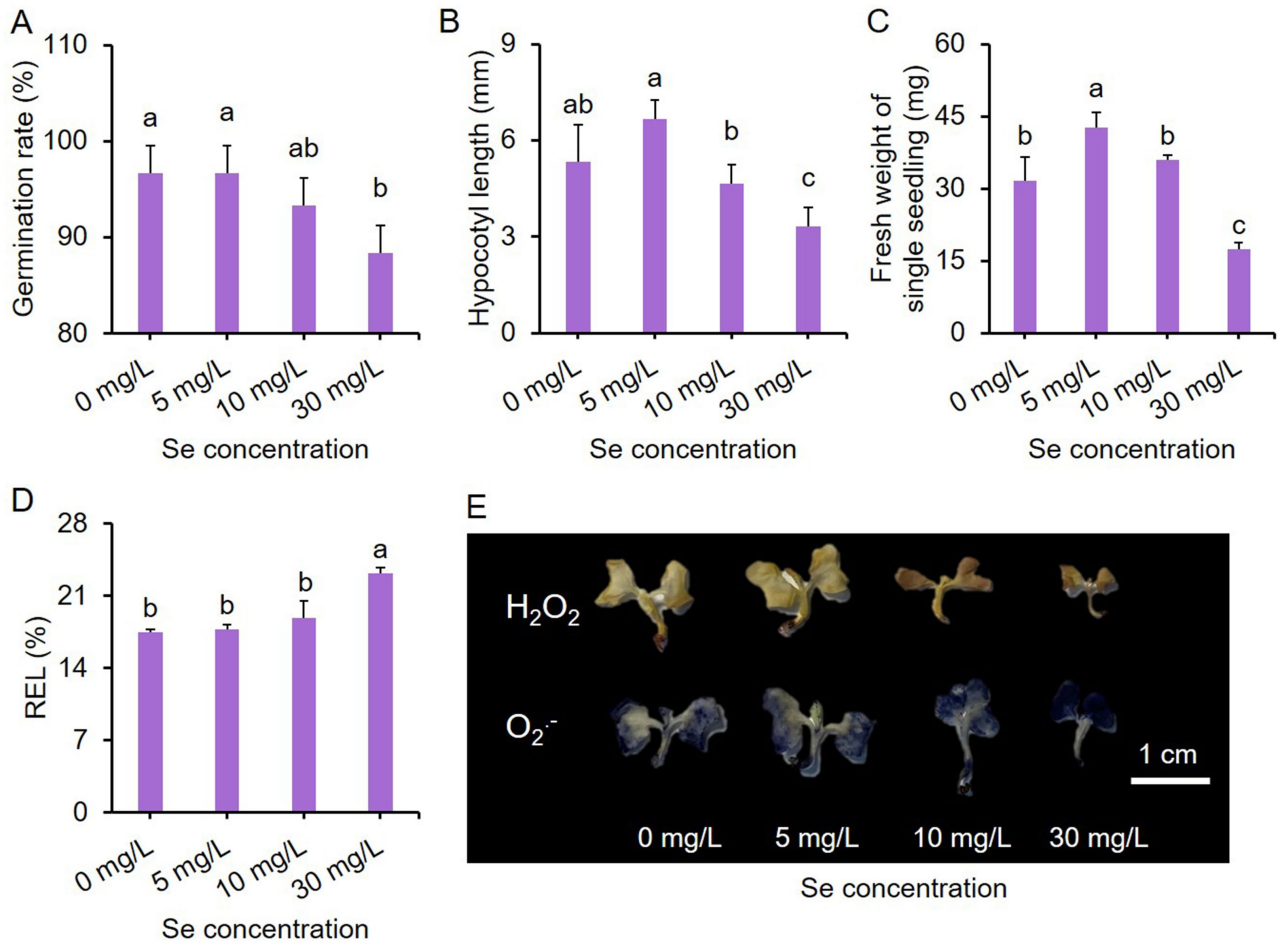
Effects of Se stress on the phenotype of purple leaf mustard young seedlings. **(A)** Germination rate of seeds under Se stress conditions; **(B)** hypocotyl length of seedlings; **(C)** fresh weight of single seedling; **(D)** relative electrolyte leakage (REL) of seedlings under Se stress conditions; **(E)** hydrogen peroxide (H_2_O_2_) and superoxide anion (O_2_^-.^) staining in seedlings. Seedlings were incubated in different concentrations of sodium selenite solutions (0, 5, 10, and 30 mg/L) for 7 days. The data is presented as means ± standard error from three replicates. Statistical differences (*p* < 0.05) are denoted by different letters above the bars.

The growth performance of seedlings subjected to different intensities of Se stress treatments (final concentrations of Se at 0, 5, 10, and 30 mg/L) is depicted in Supplementary Figure S4, revealing that the 30 mg/L Se treatment led to noticeably smaller biomass for the seedlings. Se concentrations below 30 mg/L did not hinder seed germination rates, whereas the 30 mg/L Se treatment significantly reduced germination rates (Figure 6A).

The impact of different concentrations of Se treatments on hypocotyl length (Figure 6B) and fresh weight of single seedling (Figure 6C) showed similar trends overall. In comparison to the control, the 5 mg/L Se treatment enhanced hypocotyl length and fresh weight, while the 30 mg/L Se treatment decreased them. The hypocotyl length and fresh weight of the 10 mg/L Se treatment were comparable to those of the control. Notably, only the 30 mg/L Se treatment resulted in a significantly higher relative electrolyte leakage (REL) compared to the other treatments (Figure 6D).

H_2_O_2_ and O_2_^.-^ are two primary ROS in plants during abiotic stress (47). We also analyzed the accumulation of H_2_O_2_ and O_2_^.-^ in seedlings exposed to Se stress conditions. Low concentrations of Se treatments led to a slight increase in the levels of these two ROS, while the 30 mg/L Se treatment resulted in higher ROS accumulation (shown by a darker color) compared to the control group (Figure 6E). These results together suggest that the developmental stage of the plant may also influence Se sensitivity, with young seedlings being more sensitive to Se stress. Further investigations are required to compare Se enrichment efficacy of distinct application methods.

## Discussion and conclusion

Both Se and anthocyanins play essential roles in promoting human health (48, 49). For instance, Se exhibits a broad spectrum of pleiotropic effects, including antioxidant and anti-inflammatory properties, as well as its role in the production of active thyroid hormones (50). Similarly, anthocyanins possess potent oxygen radical scavenging abilities and are known to contribute to the delay of aging in humans (19). Plants have the ability to absorb Se from the soil, making the cultivation of vegetables in Se-rich soils a viable and safe strategy for biofortifying the nutritional quality of food with this essential nutrient. In this study, sodium selenite was applied to the roots of purple leaf mustard, leading to a significant increase in Se enrichment in tissues, and Se contents increased with increasing treatment concentrations (Figures 3A–C). More importantly, Se treatment markedly boosted the anthocyanin content in leaf mustard, resulting in a deepening color on the leaf surface (Figure 2). These results suggest that preharvest Se treatment not only enhances the levels of Se and anthocyanins in vegetable tissues, but also improves the visual appeal of leaf mustard leaves, making them more attractive to consumers. Anthocyanins are primarily produced through the phenylpropanoid pathway, with their biosynthesis relying on the activity and gene expression of several key enzymes such as PAL (51). A widely targeted metabolome analysis revealed that preharvest application of Se significantly enhanced the contents of several phenolic acids, and meanwhile upregulated the expression of key genes in the phenylpropanoid pathway such as *C4H*, *COMT*, *CHS*, and *FLS* (36). In this study, Se treatment enhanced PAL activity and notably upregulated the expression of 12 genes (including *PALs*, *4CLs*, *CHSs*, *CHIs*, *F3Hs* and *F3’H3*) responsible for anthocyanin biosynthesis in leaf mustard (Figure 4), demonstrating positive roles of Se treatment on anthocyanin biosynthesis at gene level. These results further suggest that Se treatment promotes anthocyanin biosynthesis by directly activating the expression of genes responsible for the process.

The TSS, GSH, ascorbic acid, FAA, phenolics and carotenoids are essential nutrients that offer significant health benefits (52, 53). In this study, Se treatments were found to increase the levels of TSS, GSH, ascorbic acid, FAA, total phenolic compounds and soluble proteins in purple leaf mustard (Figures 2, 3), indicating that, with the exception of Se and anthocyanins, preharvest Se treatment also positively influenced the levels of other nutrients. These results align with similar results observed in pepper fruit (54) and radish sprouts (23). Besides their nutritional benefits, TSS, FAA and soluble proteins serve as crucial osmotic substances in plants (55), while GSH and ascorbic acid functions as important non-enzymatic antioxidants (56). It could be reasonably speculated that the evaluated these substances can help protect leaf mustard from oxidative damage induced by Se stress.

Although Se is not considered essential for plants, it is recognized as a beneficial element that can promote plant growth (57). However, many studies have shown that the beneficial effects of Se application are constrained by its concentration. For instance, in apple plants, lower Se treatments (6, 9, and 12 μM) have been shown to enhance plant growth, whereas higher concentrations of Se (>12 μM) inhibit growth, particularly in root development (11). In our current study, lower Se concentrations, particularly at 10 mg/kg or 5 mg/L, significantly enhanced the growth of leaf mustard plants and seedlings. However, higher Se concentrations (30 mg/kg or 30 mg/L) markedly hindered plant (Table 1) and seedling growth (Figure 6). Similar results have been observed in cabbage, another leafy vegetable of the *Brassica* family (15). These results suggest that the impact of Se preharvest treatment on the growth of leaf mustard is dependent on the treated concentration, with only an appropriate concentration leading to growth promotion. Furthermore, many factors, including growth conditions, influence the growth and Se absorption in plants (58). In this study, leaf mustard seedlings were grown in a growth container with strictly controlled relative humidity, light, and temperature. Further investigations are necessary to clarify how and whether growth conditions affect the growth and Se absorption in purple leaf mustard under Se stress conditions.

Plant growth and development under abiotic stress conditions are closely associated with the antioxidant system (59). In Se-enriched plants, Se could induce cellular ROS and thus influence cell death via the apoptosis mechanism at toxic concentrations (6). In this study, a high Se concentration (30 mg/kg or 30 mg/L) resulted in significantly higher H_2_O_2_ and MDA contents (Figure 5), as well as REL and O_2_^.-^ (Figure 6), indicating that a high Se concentration triggered oxidative damages on mustard tissues. This was attributed to the failure of ROS scavenging, as the 30 mg/kg Se treatment significantly reduced the activities of several antioxidant enzymes including APX, CAT and POD (Figure 5). Of greater importance, the 10 mg/kg Se treatment simultaneously enhanced antioxidant enzyme activity but had lower H_2_O_2_ and MDA contents compared to the high Se concentration, and promoted growth. This suggests that the enhanced antioxidant activity could promptly scavenge ROS, thereby avoiding Se-triggered oxidative damage and guaranteeing normal growth. This further highlights the importance of the antioxidant system for leaf mustard under Se stress. These results are consistent with results observed in different plant species, such as cabbage (60) and mushroom (61), further demonstrating the dual effects of Se treatment on antioxidant enzymes. In addition to triggering oxidative stress, H_2_O_2_ also serves as a crucial signaling molecule in plant stress responses and has the potential to stimulate antioxidant activity through feedback mechanisms (62, 63). The elevated levels of H_2_O_2_ observed in low concentrations of Se treatments (Figures 5A, 6E) may play a role in inducing antioxidant activity.

In addition to the antioxidant system, plant biomass primarily relies on photosynthesis (64). Previous studies have shown that Se treatment has dual effects on plant photosynthesis (23). In purple leaf mustard here, low levels of Se treatments enhanced photosynthetic parameters, increased chlorophyll pigment content, and improved chlorophyll fluorescence (Figure 1). A significant linear correlation between Se content and photosynthesis rate, as well as chlorophyll fluorescence, was observed in rice following Se treatment (65). It was observed that the 30 mg/kg Se treatment did not reduce total chlorophyll content or chlorophyll fluorescence (*Fv/Fm*) compared to the control (Figure 1). These results suggest that Se application can enhance the rate of photosynthesis in purple leaf mustard by promoting chlorophyll production, resulting in increased photosynthetic activity. However, the 30 mg/kg Se treatment led to a decrease in biomass compared to the control group (Table 1). Additionally, only the 10 mg/kg Se treatment enhanced the ratio of total chlorophyll and anthocyanin content, while the 30 mg/kg Se treatment significantly reduced this ratio compared to the control (Supplementary Figure S2). Since anthocyanins are primarily accumulated in the epidermal cells of *Brassica* vegetable leaves (29), and high Se concentrations also increased anthocyanin content (Figure 2E), the ratio of chlorophyll to anthocyanin in leaves appears to affect photosynthetic efficiency. This may explain why high concentration of Se treatment reduced the photosynthesis rate without reducing chlorophyll content.

**Figure 7:**
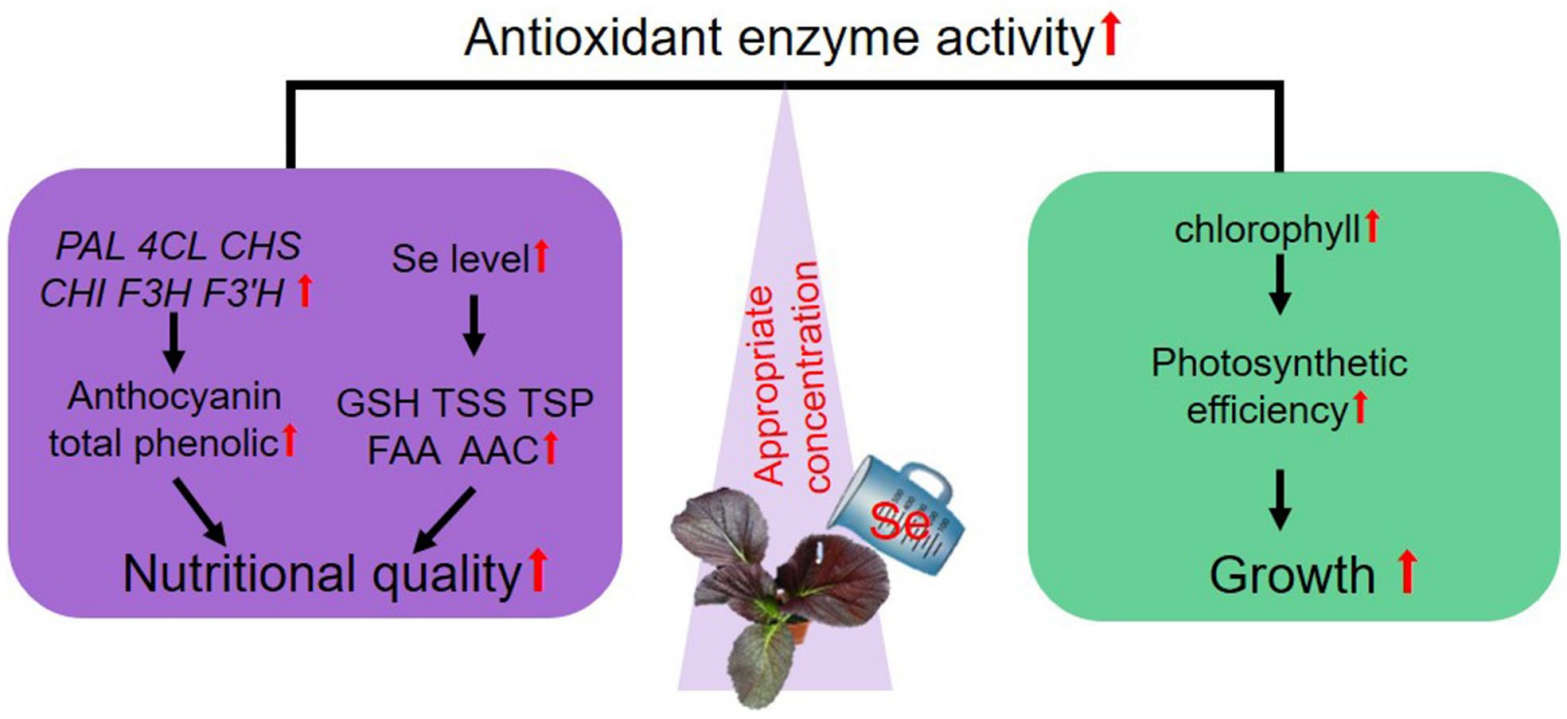
A proposed model illustrating how suitable Se treatment balances the growth and nutritional quality of purple leaf mustard. “↑” denotes a reinforcement or heightened impact by Se treatment.

In conclusion, preharvest Se treatment with varying concentrations had distinct effects on the growth and nutritional quality of leaf mustard. Treatment at 10 mg/kg balanced the growth and anthocyanin biosynthesis, suggesting the application of Se preharvest at a proper concentration might be a promising method for anthocyanin fortification in fresh food products. Based on the results and discussion presented above, we proposed a model to suggest how suitable concentration of Se preharvest treatment balances the growth and the nutritional quality of purple leaf mustard (Figure 7). A 10 mg/kg Se treatment boosted growth by improving photosynthetic efficiency and increased the contents of key nutrients, particularly anthocyanins, by upregulating the expression of key genes responsive for their biosynthesis. Moreover, Se treatment enhanced antioxidant activities, which might play a crucial role in protecting plants from oxidative stress and improving plant health and quality. Overall, our study is the first time for studying the roles of Se in balancing the growth and anthocyanin biosynthesis in plants.

## Data Availability

The original contributions presented in the study are included in the article/Supplementary material, further inquiries can be directed to the corresponding authors.
