# Supplementary figures and images for: Preharvest sodium selenite treatments affect the growth and enhance nutritional quality of purple leaf mustard with abundant anthocyanin
Source: Front Nutr. 2024 Oct 23;11:1447084. doi: 10.3389/fnut.2024.1447084 (PMC11537877; doi:10.3389/fnut.2024.1447084)

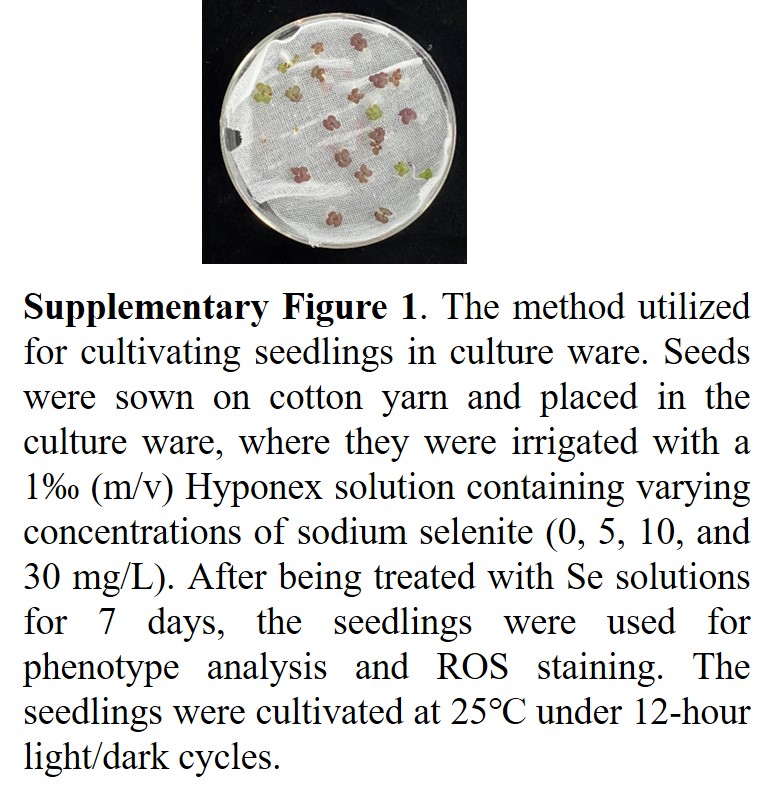

Supplement: Supplementary file 1 [file Image_1.JPEG]

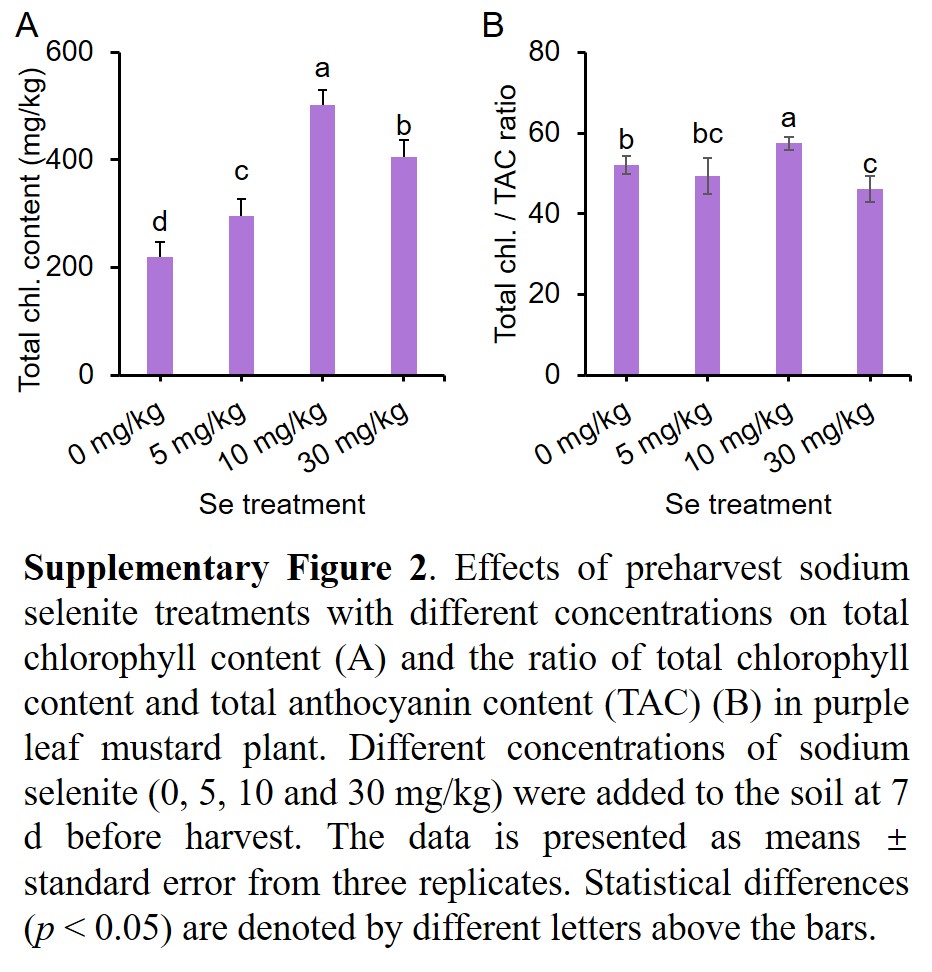

Supplement: Supplementary file 2 [file Image_2.JPEG]

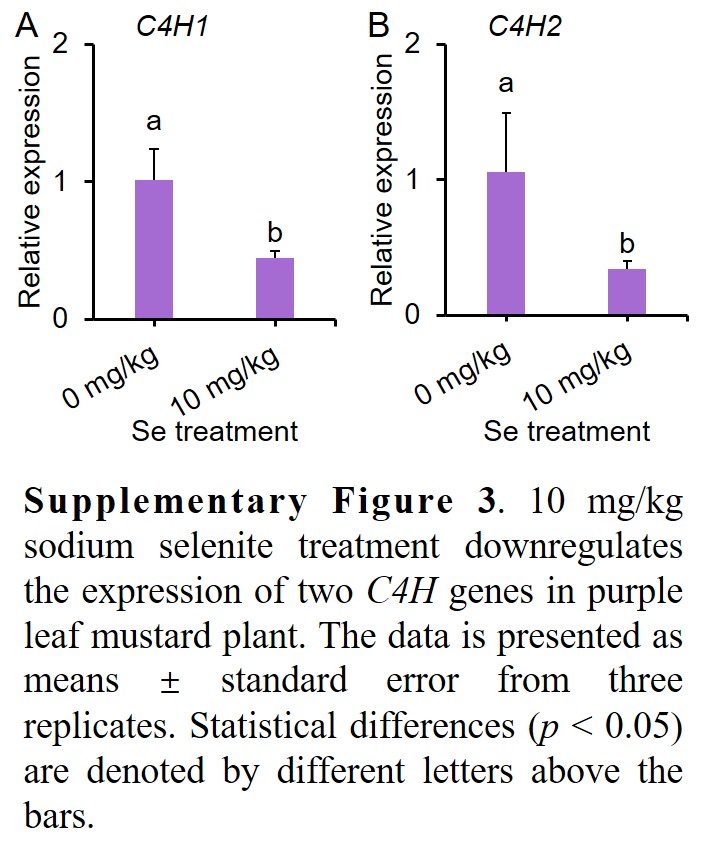

Supplement: Supplementary file 3 [file Image_3.JPEG]

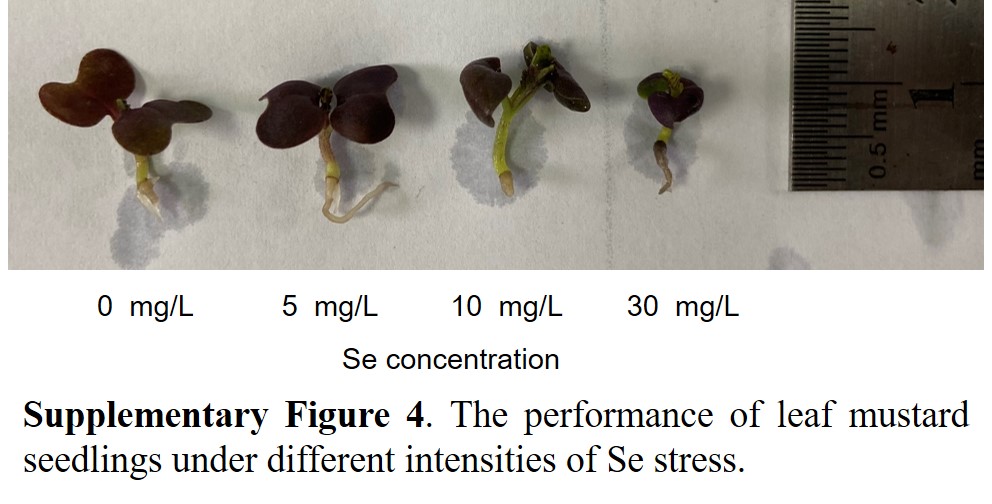

Supplement: Supplementary file 4 [file Image_4.JPEG]
