# Supplementary material for: Preharvest sodium selenite treatments affect the growth and enhance nutritional quality of purple leaf mustard with abundant anthocyanin
Source: Front Nutr. 2024 Oct 23;11:1447084. doi: 10.3389/fnut.2024.1447084 (PMC11537877; doi:10.3389/fnut.2024.1447084)
Supplement: Supplementary file 5 [file Table_1.DOC]

**Supplementary Table 1.** Primer sequences used for qRT-PCR analysis.

| Genes | Forward primer sequence (5'-3') | Reverse primer sequence (5'-3') | PCR product size (bp) |
| --- | --- | --- | --- |
| *BjActin* | CACGGTGTTGTGAGCAACTG | GCGATACGGAGCTCGTTGTA | 74 |
| *Bj4CL4* | ATACACGCAGGCTGAGATCG | CGCAAACTATCAGAACGCCG | 122 |
| *BjCHS1* | CGTGTTTGATTTCCAGCCTGT | GGTGATCCAGCCATCCAACA | 141 |
| *BjCHI1* | TCTCCTCTCACCGTTCGTCT | GCAACGGATTCGGAGCTAGA | 73 |
| *BjCHI3* | TGGTGAAATCCGCAGCTTCT | CGAGCAAGGAGAGCGTTGTA | 123 |
| *BjF3'H1* | CCAGTCGTACTATTCCCGGC | GCAGCAATGGACACACCATC | 103 |
| *BjF3H3* | CTTTTTGCTCCGGGGAAAGC | CCTCGCCTGTAGATAGCACG | 73 |
| *BjCHS2* | CGACACGTCCTTAACCACCA | GCAATCAAGCCGAGCAACTT | 108 |
| *BjF3'H1-like* | AGTGCCTCCGTTCTCCCTAT | AGCTTCGGGGTTTTTGGTCA | 132 |
| *BjPAL1* | GGCTGATCTCGAGTCGGAAG | GCAACCAAGTAGGTCGTGGA | 73 |
| *Bj4CL2* | ATTCACACGTCGCATGCTCA | GTGGACAGCACCTACAGCAA | 85 |
| *BjF3H1* | AATGGCGAGCACCTGAAAGT | ACCAGCGATTCTGGAGCAAA | 76 |
| *BjPAL like* | TCATACCGCTCGCTTGACTC | ATCGGAACCTTGGACGGAAC | 159 |
| *BjC4H1* | GGGGAGAGAAGCAGGTTGAC | AGCCTCTAAGGAACGGCCTA | 82 |
| *BjC4H2* | AGGCCGTTCCTTAGAGGCTA | GCGCATTTCAATCCATCGCT | 140 |
